# Per Aspenberg, 1949–2018

**DOI:** 10.1080/17453674.2018.1491747

**Published:** 2018-07-23

Per Aspenberg, 1949–2018


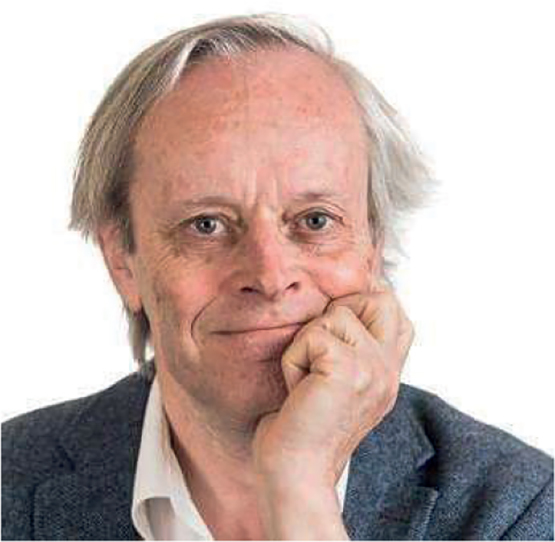


Professor Per Aspenberg has sadly passed away. He was for many years one of the most influential personalities in the Swedish orthopedic community and combined a brilliant intellect with a constant curiosity and innovative ability. He began his career as an orthopedic surgeon in Västerås but was from early on interested in research, which attracted him to the academic environment at Lund University, where he soon became a well-respected scientist with a primary focus on bone healing and metabolism. After having distinguished himself as a successful researcher with an established international reputation he was in 2001 appointed Professor of Orthopedics at Linköping University, his home town. Here he continued his work in the field of bone and tendon healing by taking charge of the local research laboratory, which he revitalized. He was the principal mentor of almost 30 PhD projects, several of which have had a significant impact on orthopedic science.

Aspenberg’s research spanned wide fields, among them bone metabolism, epidemiology, and fracture treatment. He invented the “Bone chamber,” successfully used for numerous bone metabolism experiments. He could show in animal and clinical studies that PTH advances fracture healing. He was early in identifying the substantially increased risk of atypical fractures associated with bisphosphonate use but could also show that bisphosphonates could be used to decrease the risk of loosening of joint prostheses. One of his hobbyhorses was to question the benefit of some fracture surgery. He applied an American questionnaire used to identify unsuitable pilots to show that the propensity to do (unnecessary) surgery increased with what was identified by the questionnaire as macho features (not good in pilots and perhaps not in surgeons…).

His never-ending enthusiasm for science and ability to inspire his colleagues and coworkers was always sincerely appreciated and he had the aptitude of conveying a positive atmosphere to whatever gathering in the department. Among other achievements, his CV comprises over 300 publications in medical journals (1 in this issue of Acta, pp 457–461) and although the majority of his substantial scientific production was based on laboratory work, he took part in many clinical studies and was constantly involved and knowledgeable in everyday clinical practice. Being open-minded and interested in all fields of orthopedics he was always the perfect discussion partner.

Aspenberg had been co-editor of *Acta Orthopaedica* since 2005 and helped many authors to further improve already good manuscripts. He was just as good at explaining to authors why their manuscripts should not be published, but without offending them.

He enjoyed music and art and was a talented musician as well as a keen craftsman in wood. The annual meeting of the Swedish Orthopaedic Society will truly miss his ingenious wooden sculptures that were always displayed at the session of “Art in Orthopaedics.” He was a sailor, and often alone in the boat (see https://youtu.be/voAqdCrSnu8).

Even in the last days of his life he stayed engaged in research and only a few weeks before his demise he published an article questioning the role of the placebo effect, typical of his commitment to challenge established concepts that he felt had been misinterpreted or misused.

The orthopedic community has lost one of its most colorful, influential and important individuals and he is sincerely missed.

**Lars Adolfsson and all Editors of Acta Orthopaedica**

